# Fabrication of High-Performance Colorimetric Membrane by Incorporation of Polydiacetylene into Polyarylene Ether Nitriles Electrospinning Nanofibrous Membranes

**DOI:** 10.3390/nano12244379

**Published:** 2022-12-08

**Authors:** Pan Wang, Xidi Liu, Yong You, Mengxue Wang, Yumin Huang, Ying Li, Kui Li, Yuxin Yang, Wei Feng, Qiancheng Liu, Jiaqi Chen, Xulin Yang

**Affiliations:** 1 School of Mechanical Engineering, Chengdu University, Chengdu 610106, China; 2 Key Laboratory of General Chemistry of the National Ethnic Affairs Commission, School of Chemistry and Environment, Southwest Minzu University, Chengdu 610041, China; 3 Research Branch of Advanced Functional Materials, School of Materials and Energy, University of Electronic Science and Technology of China, Chengdu 610054, China; 4 Institute for Advanced Materials Deformation and Damage from Multi-Scale, Chengdu University, Chengdu 610106, China

**Keywords:** polyarylene ether nitrile, high-performance, colorimetric sensor, polydiacetylene, electrospinning membrane

## Abstract

Polyarylene ether nitrile (PEN) is a novel high-performance engineering plastic with various applications, particularly in thermoresistance-required fields. In this study, a well-known stimuli-response polydiacetylene monomer, 10, 12-pentacosadiynoic acid (PCDA), was encapsulated within electrospun PEN nanofibers to fabricate a colorimetric membrane with satisfactory thermal and corrosion resistance. To optimize the compatibility with PCDA, two PENswith distinct molecular chains were utilized: PEN−PPL and PEN−BPA. The chemical structure and elemental mapping analysis revealed that the PCDA component was successfully incorporated into the PEN fibrous. The PCDA bound significantly better to the PEN−PPL than to the PEN−BPA; due to the carboxyl groups present on the side chains of PEN−PPL, the surface was smooth and the color changed uniformly as the temperature rose. However, owing to its poor compatibility with PEN−BPA, the PCDA formed agglomerations on the fibers. The thermal analysis demonstrated that the membranes obtained after PCDA compounding maintained their excellent heat resistance. The 5% weight loss temperatures of composite nanofibrous membranes manufactured by PEN−PPL and PEN−BPA were 402 °C and 506 °C, respectively, and their glass transition temperatures were 219 °C and 169 °C, respectively, indicating that the blended membranes can withstand high temperatures. The evaluation of application performance revealed that the composite membranes exhibited good dimensional stability upon high thermal and corrosive situations. Specifically, the PEN−P−PCDA did not shrink at 170 °C. Both composite membranes were dimensionally stable when exposed to the alkali aqueous solution. However, PEN−P−PCDA is more sensitive to OH^−^, exhibiting color transition at pH > 8, whereas PEN−B−PCDA exhibited color transition at high OH^−^ concentrations (pH ≥ 13), with enhanced alkali resistance stability owing to its nanofibrous architecture. This exploratory study reveals the feasibility of PEN nanofibers functionalized using PCDA as a desirable stimulus-response sensor even in high-temperature and corrosive harsh environments.

## 1. Introduction

Poly(arylene ether)s are well-known as a type of famous high-performance engineering plastic, with examples such as polyarylene ether ketone, polyarylene ether sulfone and polyarylene ether nitrile (PEN), due to their backbone structures consisting of alternating aromatic rings and ether bonds. The combination of hardness and softness endows poly(arylene ether)s with inherent chemical, irradiation and mechanical stability, as well as outstanding resistance [1,2,3]. PEN, which possesses a feature nitrile group (−CN) attached to the side chain, has been used commercially as a high-performance thermoplastic material since 1986 [4]. The pendant–CN distinguishes PEN from the poly(arylene ethers) family owing to its superior thermochemical resistance and molding workability [5]. In recent years, PEN has attracted attention for high-tech engineering applications, such as matrix-reinforced composites in the aerospace manufacturing industry, high-thermoresistance capacitors in electronic devices, dimensional- and heat-stabilized proton exchange membranes in fuel cells, robust filtration membranes in sewage treatment in harsh environments and novel durable stimuli-responsive fluorescent polymers in heavy metal detection [6,7,8,9,10]. The aforementioned studies pave the way for high-performance PEN applied in various fields.

As a typical conjugated polymer, polydiacetylene (PDA) has attracted considerable interest in the field of smart detection owing to its unique color response property of changing from blue to red when exposed to external stimuli, such as pH [11], metal ions [12,13], organic solutions [14], CO_2_ [15], ammonia [16,17], biogenic amine gas [18], bacteria [19] and heating [20,21,22,23]. PDA is typically produced via the 1,4-addition polymerization of diacetylene monomer (DA) via UV or γ-ray irradiation in the absence of any other initiator or catalysis [24]. According to the current study, the as-formed PDA is arranged in a planar structure in a highly ordered conjugated state and appears blue under visible light. However, upon the above-mentioned stimulation, the C−C bond on the backbone rotates and distorts the π-conjugated main chain, causing a blue shift in the absorption spectrum, so that it appears red [23,25]. For practical application scenarios, PDA-based sensors have been fabricated as solid-state materials, such as paper-based film, nanofibers/microfibers and cast/spin-coated membranes, with easy handling, cost-effectiveness and robust performance when compared to powder, vesicles and nanoparticles [26,27,28,29]. Hereinto, nanofibers/microfibers produced via electrostatic spinning usually exhibit a mesh-like structure resulting from the collection of polymer jets under the influence of an electric field [30]. These membranes have a high specific surface area, high porous structure with small pores and nanofibers/microfibers, as well as the ability for facile functionalization [10]. Up to now, electrospinning membranes have been widely applied in waste water treatment, batteries, bioengineering, sensors, etc. [31,32]. The above advantages also enable the electrospinning membrane-based sensors to be highly sensitive to various stimuli [33]. Moreover, the performance of electrospinning membranes in applications is affected by the substrate material selected. Valdez et al. [18] opted to use polyethylene glycol blended with PDA. The melting point of polyethylene glycol is approximately 64 °C, but the temperature shift of PDA is typically above 60 °C; therefore, these nanofibrous membranes cannot be used for temperature sensing. Alam et al. [34] employed polyethylene oxide, but its low melting point is at 62 °C and it has insensitivity to temperature changes. In contrast, Mapazi et al. [35] blended polyacrylonitrile with PDA and successfully obtained nanofibrous membranes that can be used for temperature response because polyacrylonitrile’s thermal decomposition temperature is above 230 °C. Notably, when the material is prepared as nanofiber/microfiber for application, some of its properties, such as heat resistance and corrosion resistance, are diminished compared to when the materials are prepared with the bulk and casting film. Consequently, the performance of a polymer candidate for an electrospinning-based PDA sensor should be more than adequate.

**Scheme 1:**
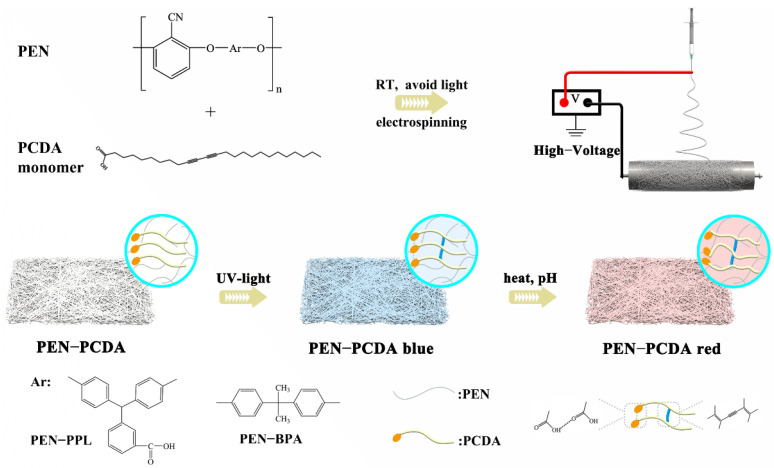
The schematic illustration of the fabrication of PEN−PCDA composite colorimetric nanofibrous membrane.

In this study, two types of PEN, namely phenolphthalin (PEN−PPL) and bisphenol A (PEN−BPA), were chosen for blending with a typical PDA, 10,12-dipentadecyldiyne carboxylic acid (PCDA). PEN−PPL contains carboxyl groups on the side chain and is typically used as a reactive polymer for functionalization [36,37]. Meanwhile, commercial PEN−BPA synthesized with bisphenol A (BPA) has a higher thermal decomposition temperature and mechanical strength, as well as corrosive stability [38]. Moreover, both selected PENs possess good electrospinning ability. Then, the composite membranes combined with PEN and PCDA (PEN−PCDA) were fabricated via electrospinning technology (see Scheme 1), and their stimulus-response performance as well as thermal and alkali resistance were investigated in an effort to develop a PDA-based colorimetric sensor with comprehensive performance benefitting from high-performance PEN.

## 2. Materials and Methods

### 2.1. Materials

PEN−PPL was synthesized in our laboratory as per the previous report [37]. Meanwhile, PEN−BPA was purchased from Sichuan Energy Investment Chuanhua New Material Technology Co., Ltd. (Chengdu, China) since it is one of the commercial types of PEN. It was used after purification with dilute hydrochloric acid and deionized water to remove residual organic solvent and catalyst. The employed typical PDA monomer is 10, 12-pentacosadiynoic acid (PCDA) (98%, RG), which was obtained from Shanghai Titan Technology Co. (Shangai, China), Ltd. N, N-dimethylformamide (DMF) (AR) and NaOH (AR) were purchased from Chengdu Kelong Chemical Co., Ltd. (Chengdu, China) and used directly without further purification.

### 2.2. Synthesis

#### 2.2.1. Preparation of Nanofibrous Membranes

PCDA was dissolved in DMF at 0.1 g/mL in advance. A certain amount of PEN−PPL and PEN−BPA were added to separate brown glass bottles. PCDA powder was added to the DMF, then the PEN and PCDA solutions were mixed, making sure the mass ratio of PEN: PCDA was 5:1. Then, to obtain a solution with enough viscosity for spinning, DMF was supplemented until the concentration reached 0.50 g/mL for PEN−PPL and 0.17 g/mL for PEN−BPA, due to their different electrospinning abilities. After that, a magnetic stirrer was used to stir the dissolution at room temperature while avoiding light for 6 h to obtain a homogeneous solution.

Subsequently, the nanofibrous membranes were blended with a mixture of PENs and PCDA via electrostatic spinning. The well-mixed spinning solution was transferred into a 5 mL syringe with a stainless-steel needle (0.3 mm inner diameter). Then the syringe was loaded in an electrostatic spinner with the tip of the needle 15 cm from the collector. The injection speed and rotation speed were set as 0.001 mm/s and 300 rpm/min, respectively, while spinning voltages were adjusted at 25 kV for PEN−PPL and 20 kV for PEN−BPA. An aluminum foil was employed as the collection substrate. To protect the photosensitive mixtures and as-prepared fibers from environmental heat and light, the experiment of electrospinning was performed under room temperature and avoiding light. After the spinning process was completed, the membranes were placed in a dark oven to dry at room temperature for more than 2 days to ensure complete volatilization of DMF solvent. Then, the well-prepared blended membranes were taken off from the aluminum foil and ready for subsequent processing. In addition, the pure PENs’ electrospun fibers were fabricated via a similar strategy and conditions with no inclusion of PCDA. The synthetic scheme of composite membranes is illustrated in Scheme 1.

#### 2.2.2. Cross-Linking of Membranes

The as-prepared membranes appeared white in color and were cross-linked into a highly ordered conjugated state, which appeared blue in color, for the subsequent stimulus-response. Then, the white membranes were stored in a dark box employed with a UV-lamp at room temperature. Each side of the membranes was irradiated with 254 nm UV light for 2 min to make sure the color changed blue completely until it no longer darkened. The corresponding cross-linked blue nanofibrous membranes were named PEN−P−PCDA and PEN−B−PCDA.

### 2.3. Characterization and Instruments

Fourier transform infrared spectrometer (FT−IR) spectra were obtained using a Thermo Nicolet IR200 (Thermo Fisher Scientific, Waltham, MA, USA) over the range of 500−3000 cm^−1^. Raman spectra were obtained by testing at 532 nm over the range of 1000−3000 cm^−1^ using a Thermo Scientific DXR, Thermo Fisher Scientific, Waltham, MA, USA. The morphology of the membranes before and after heating were investigated using emission scanning electron microscopy (ZEISS GeminiSEM 300, Carl Zeiss, Oberkochen, Germany). The elemental distribution was performed using transmission electron microscopy (JEM−F200, JEOL, Tokyo, Japan). The diameter distribution of the fibers was calculated using a Nano Measurer. The change of absorbance of membranes was tested using a UV-vis spectrophotometer (U−1901, Beijing Persee, Beijing, China) over the range of 400−700 nm. The thermal decomposition curve of the composite membranes was studied using a TGA Q50 (TA Instruments Inc., New Castle, DE, USA). The samples were heated from room temperature to 800 °C with the heating rate controlled at 20 °C /min and the flow rate at 60 mL/min in an N_2_ atmosphere. Meanwhile, the glass transition temperature of composite nanofibrous membranes was studied via differential scanning calorimetry on a DSC Q100 (TA Instruments Inc., New Castle, DE, USA). The samples were heated from room temperature to 280 °C with the heating rate controlled at 10 °C /min and the flow rate at 50 mL/min in an N_2_ atmosphere.

### 2.4. Thermal and pH Response Measurements

The cross-linked composite membranes were cut into a square (1 × 1 cm) and heated on a heating plate for 5 s, where it was observed that the membrane changed from blue to red. The heat-treated nanofibrous membrane was named after the temperature applied. (e.g., PEN−P−PCDA−RT means the membrane was as prepared at room temperature (RT) with no post heat treatment. PEN−P−PCDA−80 °C means the membrane was exposed to 80 °C). For OH^−^ response testing, the cross-linked membranes were added to a beaker full of NaOH aqueous solution with certain pH (8−14).

## 3. Result and Discussion

### 3.1. Chemical Structure and Morphology Analysis of Prepared PEN-PCDA

#### 3.1.1. Chemical Structure Analysis by FT−IR Spectra

**Figure 1:**
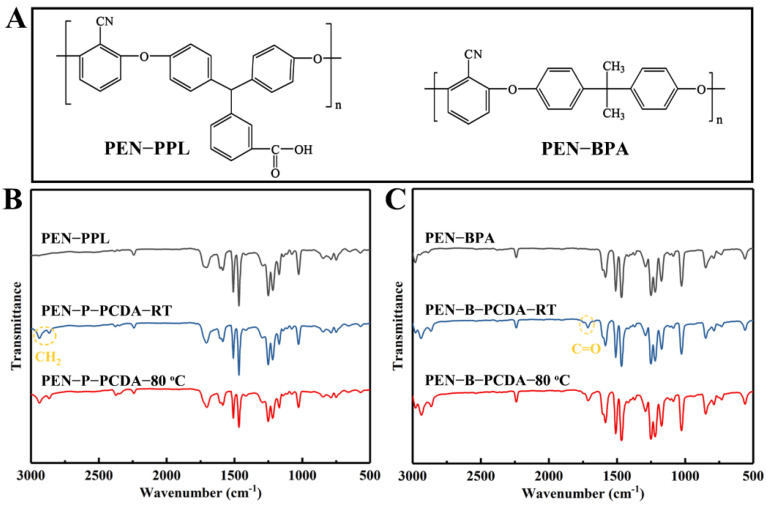
Chemical structures of PEN−PPL and PEN−BPA (**A**), FT−IR spectra of corresponding PENs and blended nanofibrous membranes at room temperature and after exposure to thermal stimulus (80 °C), PEN−P−PCDA (**B**) and PEN−B−PCDA (**C**).

The chemical structure analyses of the employed raw materials (PENs and PCDA), as well as the fabricated blends (PEN−PCDA) were measured using an FT−IR spectrometer before and after heat stimulation. Figure 1A presents the structural formulas of PEN−PPL and PEN−BPA. The difference between the two polymers, aside from the typical ether bond and cyanophenyl ring of PEN, is ascribed to the different bisphenol monomers used. PEN−PPL employs a phenolphthalein monomer, which contains carboxyl groups, whereas PEN−BPA employs bisphenol A, containing two methyl groups. FT−IR curves, as shown in Figure 1B,C, can differentiate between the two PENs. It can be seen that the spectra of PEN−PPL and PEN-BPA are relatively similar, with 2240 cm^−1^ from the stretching vibration peak of cyano (−CN), 1610−1465 cm^−1^ from the benzene ring’s vibration absorption peaks and the absorption peak near 1024 cm^−1^ from the ether band (−O−)’s asymmetric stretching vibration peak [9]. In contrast, the PEN−PPL peak at 1710 cm^−1^ is the vibrational absorption peak of the carbonyl group (C=O), which is not present in the PEN−BPA spectrum [37]. The FT−IR curves of PEN−PCDA then merge with the characteristic peaks of PCDA and the two PENs after the blending–spinning process. CH_2_ (2865 and 2937 cm^−1^) and COOH (1714 cm^−1^) have obvious feature peaks belonging to PCDA of PEN−P−PCDA at room temperature in Figure 1B and PEN−B−PCDA at room temperature in Figure 1C [23]. The molecular structures of PCDA including that of PCDA monomer, crosslinked states before and after exposure to external stimulus and FT−IR curves are shown in Figure S1 (see Supplementary Materials document). Meanwhile, after exposure to 80 °C, the FT−IR curves of both blended nanofibrous membranes show no significant displacement of the characteristic peaks corresponding to each group (see Figure 1B,C). The above results indicate that PCDA was successfully blended with PENs for subsequent investigations.

#### 3.1.2. Morphology Analysis via SEM and TEM

**Figure 2:**
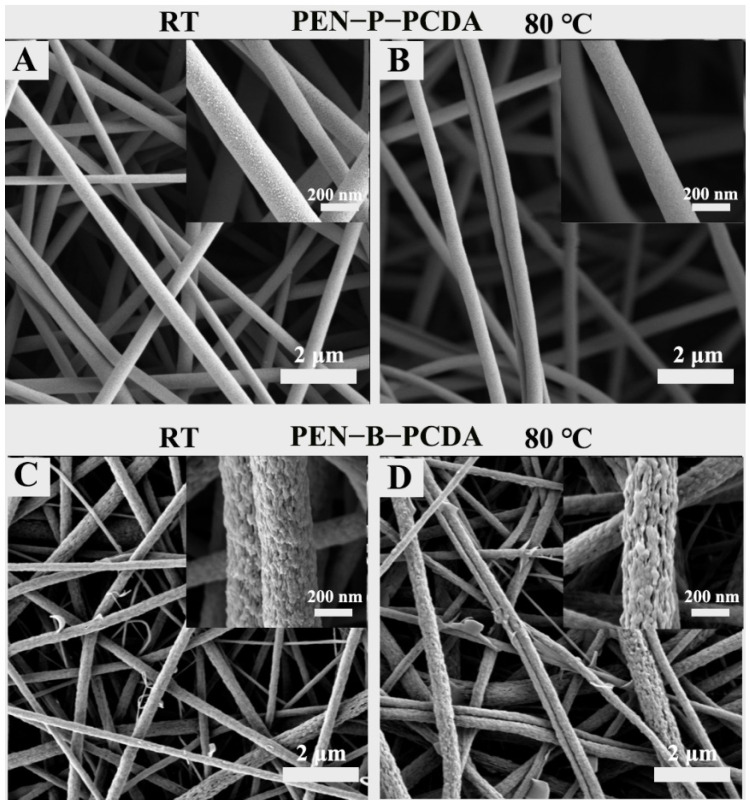
SEM images of PEN−P−PCDA and PEN−B−PCDA nanofibrous membranes at room temperature (**A**,**C**) and after exposure to 80 °C (**B**,**D**), with their corresponding local magnification images (inserts).

**Figure 3:**
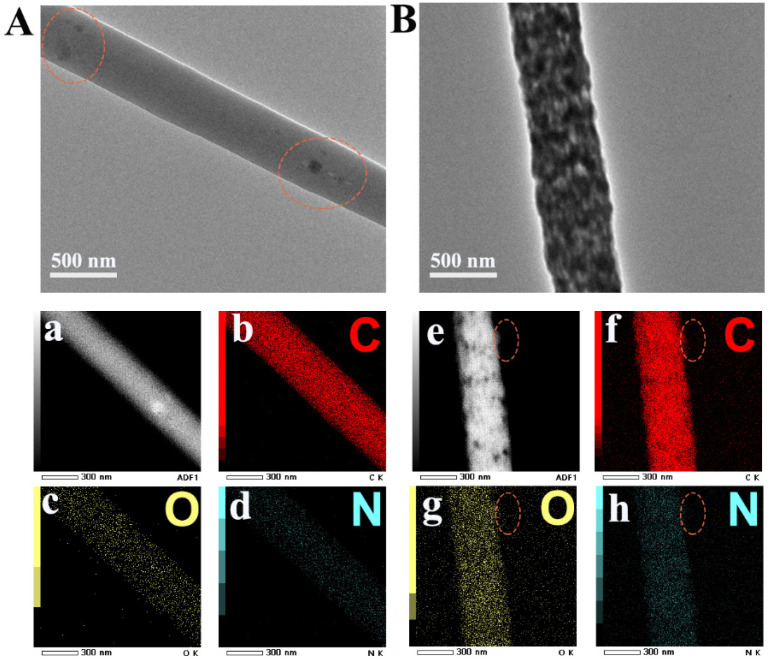
TEM images of PEN−P−PCDA−80 °C (**A**) and PEN−B−PCDA−80 °C (**B**) and elemental mappings of PEN−P−PCDA−80 °C (**a**–**d**) and PEN−B−PCDA−80 °C (**e**–**h**).

Figure 2 and Figure 3 show the SEM and TEM results, respectively, of as-prepared PEN−P−PCDA and PEN−B−PCDA. Owing to the addition of small molecules, certain polymers can alter the viscosity of the spinning solution, thus affecting the structure of the nanofibers to form beaded or discontinuous nanofibrous membranes [15]. The addition of PCDA does not affect the electrospinning capability of two composite membranes, as depicted in Figure 2. Two composite membranes continue to produce symmetrical, interconnected nanoscale fibers with large specific surface areas and porous architecture. PEN−P−PCDA has an average diameter of approximately 380 nm, while PEN−B−PCDA has an average diameter of approximately about 320 nm; neither of these values change significantly after heating at 80 °C (Figure S2). The obvious distinction between the two composite membranes is the microstructure of the fiber surface. Figure 2A depicts the presence of coarse particles on the nanofiber surface of PEN−P−PCDA. Nonetheless, the surface of the PEN−B−PCDA nanofiber is very rough and uneven, with stalactite-like lamellar structures formed by the aggregation of hybrid polymers (PEN−BPA and PCDA); see Figure 2C. This distinction is attributable to the presence or absence of carboxyl groups on the PEN chain. The carboxyl groups on PEN−PPL may form hydrogen bonds with those on PCDA [35], which also possess carboxyl groups on the molecular chain, resulting in the enhanced intercalation of PCDA and PEN−PPL during the liquid phase and the formation of homogeneous nanofibers after spinning. Poor compatibility with PEN−BPA led to stalactite-like agglomerations and also a slight amount of PCDA flake aggregates on the surface of the fiber. After heating at 80 ℃, the surface particles or protrusions of both composite nanofibrous appear to have dissolved slightly (Figure 2B,D).

In addition, TEM analysis was performed on both PEN−PCDA nanofibrous membranes to confirm the dispersion of the PCDA. As shown in Figure 3A,B, the dark areas on the surface of both single fibers represented PCDA. As a result of the good compatibility between PEN−PPL and PCDA, the PEN−P−PCDA fiber is cylindrical and uniformly loaded with a few dark spots. Contrarily, the PEN−B−PCDA fiber is dense with agglomeration structures formed by the dark PCDA. Since there is no nitrogen (N) in the chemical structure of PCDA, elemental mapping was performed on composite fibers. The distinct edge is circled in Figure 3e–h so that the element distribution can be easily identified. Carbon (C) and oxygen (O) did not significantly change in this region, whereas the N concentration decreased obversely. By carefully observing the entire mass of fibers during the occurrence of this phenomenon on the fiber’s edge, it is evident that PCDA is primarily concentrated on the fiber’s surface.

### 3.2. Thermochromic Response Behavior

**Figure 4:**
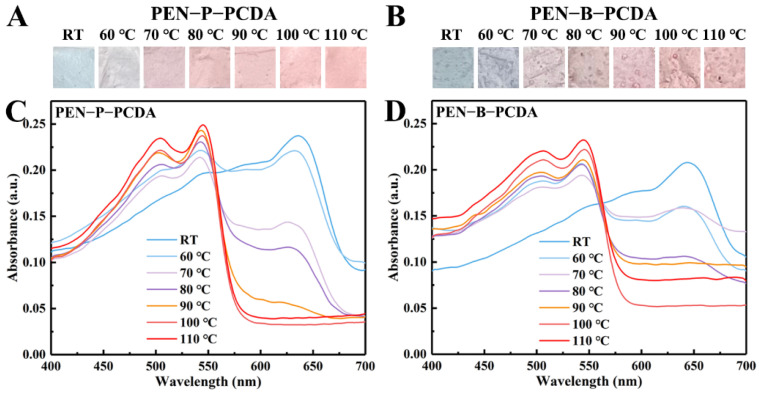
Photographic images and UV-vis spectra of PEN−P−PCDA (**A**,**C**) and PEN−B−PCDA (**B**,**D**) after exposure to various temperatures.

Figure 4A,B depict the photographic images of the PEN−P−PCDA and PEN−B−PCDA nanofibrous membranes obtained via varying the heating temperatures following the application of thermal stimulation. Owing to the planar-to-nonplanar conformational transition of the PCDA backbone, the color of the nanofibrous membranes changes from a blue phase to a red phase upon heating [17]. This is observable with the naked eye. Moreover, there will be darker regions on the membrane of PEN−B−PCDA, which may result from the poor binding of PCDA and PEN−BPA, leading to the partial aggregations. The blue-to-red colorimetric responses were further studied by recording the UV-vis spectra. According to the previous research, the blue absorption peak of PDA is typically located at ~640 nm, and when stimulated, the blue color changes to red, and the absorption peak shifts to ~540 nm [24]. In Figure 4C,D, at room temperature, the absorption peaks of PEN−P−PCDA and PEN−B−PCDA corresponding to the appearance of the blue phase peaked at 636 nm and 643 nm, respectively. In addition, there was no absorption peak at approximately 540 nm for the red phase. Through gradual heating, the color transmission of nanofibrous membranes was investigated. As the temperature rises, the blue phase absorption peak decreases gradually, while the red phase absorption peak appears gradually. The blue phase, which belongs to both membranes, disappears at 110 °C. This color switch behavior affords PEN−PCDA nanofibrous membranes temperature-induced colorimetric responsiveness range from 60 to 110 °C.

**Figure 5:**
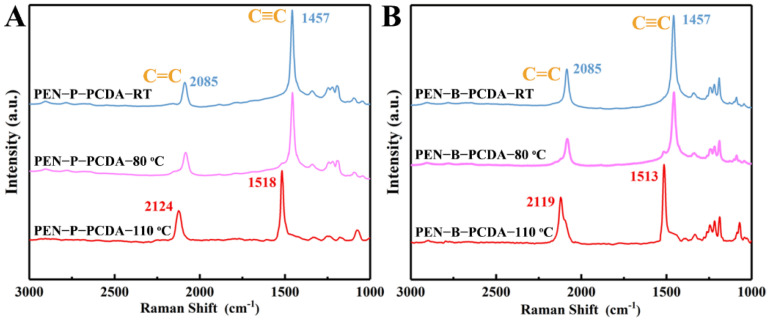
Raman spectra of PEN−P−PCDA (**A**) and PEN−B−PCDA (**B**) before and after exposure to different high temperatures (80 °C and 110 °C).

Raman measurement was also used to examine the individual structural transformation of PEN−P−PCDA and PEN−B−PCDA with increasing temperature. As shown in Figure 5A, PEN−P−PCDA−RT exhibits Raman peaks at 1457 cm^−1^ (due to C≡C stretching) and 2085 cm^−1^ (due to C=C stretching), which are characteristic of the blue phase [24]. After heating, the C≡C stretching peak redshifts from 1457 cm^−1^ to 1518 cm^−1^, and the C=C stretching Raman peak redshifts from 2085 cm^−1^ to 2124 cm^−1^. After thermal stimulation, the PCDA in the composite nanofibrous membrane undergoes a typical conformational change, according to the results. PEN−P−PCDA−80 °C demonstrates Raman peaks at all four positions, whereas PEN−P−PCDA−110 °C only demonstrates peaks at 1518 cm^−1^ and 2124 cm^−1^. This indicates that the blue conformation of PEN−P−PCDA did not undergo a complete transformation when heated to 80 °C, as the blue and red phase coexist. While the blue conformation of PEN−P−PCDA was completely transformed at 110 °C, only the Raman peak of the red phase was detected. Analyzing the Raman pattern of PEN−B−PCDA yielded a pattern identical to that of PEN−P−PCDA (Figure 5B).

### 3.3. Thermo-Resistant Performance

**Figure 6:**
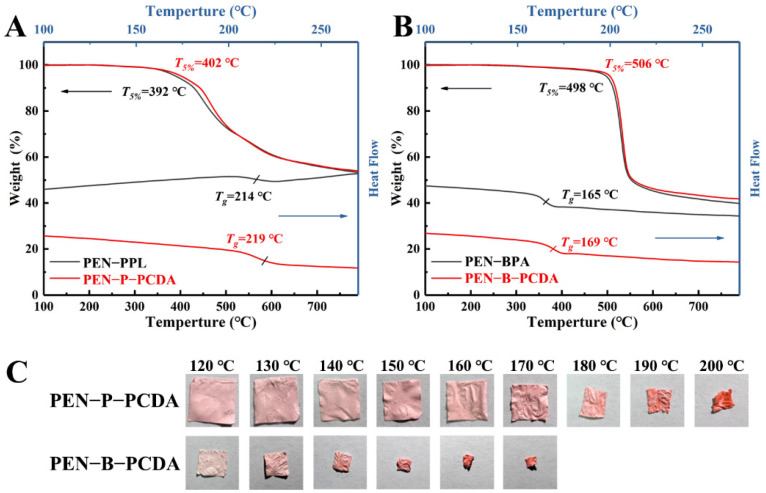
TGA and DSC curves of PEN−PPL and PEN−P−PCDA (**A**), PEN−BPA and PEN−B−PCDA (**B**), and the photographic images of nanofibrous membranes upon various high temperatures (**C**).

Thermal weight loss analysis (TGA) and differential scanning calorimetry (DSC) were used to characterize the thermo-resistant performance of PEN−PPL, PEN−BPA and their composite nanofibrous membranes blended with PCDA. As shown in Figure 6A,B, the thermal-resistance of blended membranes shows a slight improvement. The temperature at 5% thermal weight loss (*T_5%_*) increases from 392 °C to 402 °C for PEN−P−PCDA and from 498 to 506 °C for PEN−B−PCDA. The corresponding glass transition temperature (*T_g_*) increases from 214 °C to 219 °C for PEN−P−PCDA and from 165 °C to 169 °C for PEN−B−PCDA. This may be a result of the PCDA load on the nanofibers. After the initial heating, the PCDA underwent a conformational change and consumed some thermal energy, which may slightly enhance the recorded data to some extent. Moreover, similar to the previously reported improvement in thermo-resistance due to the cross-linking effect, PCDA loaded on nanofibers could bind the lapped fibers together during the crosslinking process under UV irradiation, resulting in a stronger mesh structure of the nanofibrous membranes and an increase in thermo-resistance [39]. Subsequently, PEN−PCDA nanofibrous membranes were employed to evaluate their behavior at high temperatures after cutting them into squares. As displayed in Figure 6C, PEN−P−PCDA maintains almost no shrinkage of shape up to 170 °C, followed by a dramatic structural collapse at 200 °C. In contrast, PEN−B−PCDA shrinks significantly at 120 °C. The difference in *T_g_* is attributed to this significantly different variation. The lateral methyl group on the PEN−BPA side chain is more susceptible to slippage than the benzene rings and carboxyl groups on the side chain of PEN−PPL.

### 3.4. OH^−^-Response Performance

**Figure 7:**
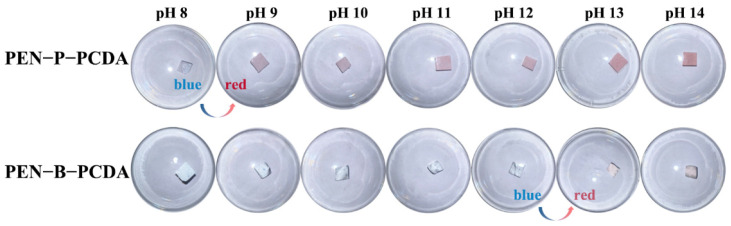
Photographic images of PEN−P−PCDA and PEN−B−PCDA nanofibrous membranes in reaction to various pH values under room temperature.

PEN is a type of high-performance engineering plastic with acid and alkali corrosion resistance properties, whereas PCDA possesses a sensitive colorimetric response to a change in pH. Typically, in an alkaline environment, hydroxide ion (OH^−^) converts the carboxylic head group to carboxylate, breaking the carboxyl hydrogen bond and rearrangement of the PCDA molecular chain, thus resulting in a blue-to-red transition [40]. In an alkaline aqueous solution (pH, 8−14), the OH^−^ response behavior of both prepared PEN−PCDA nanofibrous membranes was investigated. As the photographic images illustrate in Figure 7, both composite membranes maintain dimensional stability in a highly alkaline environment, but PEN−P−PCDA is significantly more sensitive than PEN−B−PCDA. In particular, PEN−P−PCDA nanofibrous membranes exhibit a color transition from blue to red in a solution with a pH value above 8. The color change becomes more evident as the pH rises. PCDA’s primary chain is highly conjugated in the blue phase. Furthermore, when the hydrogen bond of the PCDA is broken by OH^−^, the π-conjugated main chain rotates and distorts, causing a blue shift in the absorption spectrum and a color change from blue to red in the polymer. The stimulation intensifies as pH increases, resulting in a more pronounced color shift. Owing to their compatibility, the results indicate that PEN−PPL did not affect the pH response sensitivity of PCDA. In contrast, the color change of PEN−B−PCDA did not occur until the pH reached 13, and it was very pale. At low concentrations, OH^−^ does not affect the hue of PEN−B−PCDA. We propose that this color stability exhibited by PEN−B−PCDA can be attributed to the nanofibrous architecture. After being combined with PEN−BPA of poor compatibility, PCDA chains formed agglomerations, protecting PCDA molecules from the effect of OH^−^ in low concentrations. Therefore, these results indicate that PEN−P−PCDA nanofibrous membrane exhibits excellent thermal resistance at high temperatures and sensitivity to alkaline environments, whereas PEN−B−PCDA can detect high alkaline environments (pH ≥ 13) through the color transition.

## 4. Conclusions

This study demonstrates the fabrication of thermochromic nanofibrous membranes from stimuli–response PCDA and high-performance engineering plastic PEN. PCDA was successfully embedded and primarily dispersed on the surface of electrospun PEN fiber as evidenced by chemical structure and elemental mapping analyses. The performance of fabricated PEN−PCDA nanofibrous membranes is affected by the different polymer chains and initial properties of the two employed PEN types, PEN−PPL and PEN−BPA. In comparison, PEN−P−PCDA exhibited uniform and smooth nanofibers due to good compatibility between PEN−PPL and PCDA, whereas the PEN−B−PCDA had a rough and stalactite-like morphology attributable to agglomerations due to poor compatibility between PEN−BPA and PCDA. Both composite membranes underwent blue-to-red color transition from 60 °C to 110 °C. Moreover, the thermo-resistant evolution demonstrated that the PEN−P−PCDA nanofibrous membrane with a higher *T_g_* temperature could remain non-shrinking up to 170 °C, whereas the PEN−B−PCDA membranes collapsed just above 120 °C. In alkaline aqueous solution, both PEN−PCDA nanofibrous membranes demonstrated good dimensional stability. PEN−P−PCDA is sensitive to OH^−^, with color transition occurring at pH exceeding 8 and gradually darkening as pH increases. In contrast, PEN−B−PCDA undergoes color transition at high OH^−^ concentrations (pH ≥ 13). We propose that this alkali resistance stability is a result of the architecture of nanofibers, as the agglomerations prevent the head carboxyl group from reacting with OH^−^. Using two typical PENs, both PEN−PCDAs demonstrated their advantages in colorimetric stimulus-response behavior. The current intriguing advantages also inspire us to apply PEN−PCDAs as colorimetric sensors for volatile organic compounds in biomedicine and other fields. For instance, due to its amino group, PEN−PCDA can be further employed for biogenic amine gas, bacteria and virus detection. Moreover, it is believed that the stimulus-response behavior can be altered by modifying the chemical structure of the polymer or via blending. This exploratory study opens a way for PEN nanofibrous membranes to take advantage of thermo-resistance and corrosion resistance, etc., to be utilized in desirable high-performance stimuli-response sensors.

## Data Availability

Data are contained within the article.

## Supplementary Materials

The following supporting information can be downloaded at: https://www.mdpi.com/article/10.3390/nano12244379/s1, Figure S1: Chemical structures of PCDA at situations of monomer, blue phase and red phase (A), and the FT−IR curves of blue phase and red phase. (B); Figure S2: Statistical results of diameter of PEN−P−PCDA (A and C) and PEN−B−PCDA (B and D) nanofibers.
