# Supplementary material for: Fabrication of High-Performance Colorimetric Membrane by Incorporation of Polydiacetylene into Polyarylene Ether Nitriles Electrospinning Nanofibrous Membranes
Source: Nanomaterials (Basel). 2022 Dec 8;12(24):4379. doi: 10.3390/nano12244379 (PMC9785282; doi:10.3390/nano12244379)
Supplement: Supplementary file 1 [file nanomaterials-12-04379-s001.zip › nanomaterials-2031897-supplementary.pdf]

Supplementary Materials

Fabrication of High-Performance Colorimetric Membrane by Incorporation of Polydiacetylene into Polyarylene Ether Nitriles Electrospinning Nanofibrous Membranes

Pan Wang [1,*], Xidi Liu [1], Yong You [2], Mengxue Wang [1], Yumin Huang [3], Ying Li [1], Kui Li [1], Yuxin Yang [1], Wei Feng [1], Qiancheng Liu [4], Jiaqi Chen [1,*] and Xulin Yang [1,*]

[1] School of Mechanical Engineering, Chengdu University, Chengdu 610106, China
[2] Key Laboratory of General Chemistry of the National Ethnic Affairs Commission, School of Chemistry and Environment, Southwest Minzu University, Chengdu 610041, China
[3] Research Branch of Advanced Functional Materials, School of Materials and Energy, University of Electronic Science and Technology of China, Chengdu 610054, China
[4] Institute for Advanced Materials Deformation and Damage from Multi-Scale, Chengdu University, Chengdu 610106, China
* Correspondence: wangpan@cdu.edu.cn (P.W.); jiaqichen2014@163.com (J.C.); yangxulin@cdu.edu.cn (X.Y.); Tel.: +86-028-84616169 (P.W.)

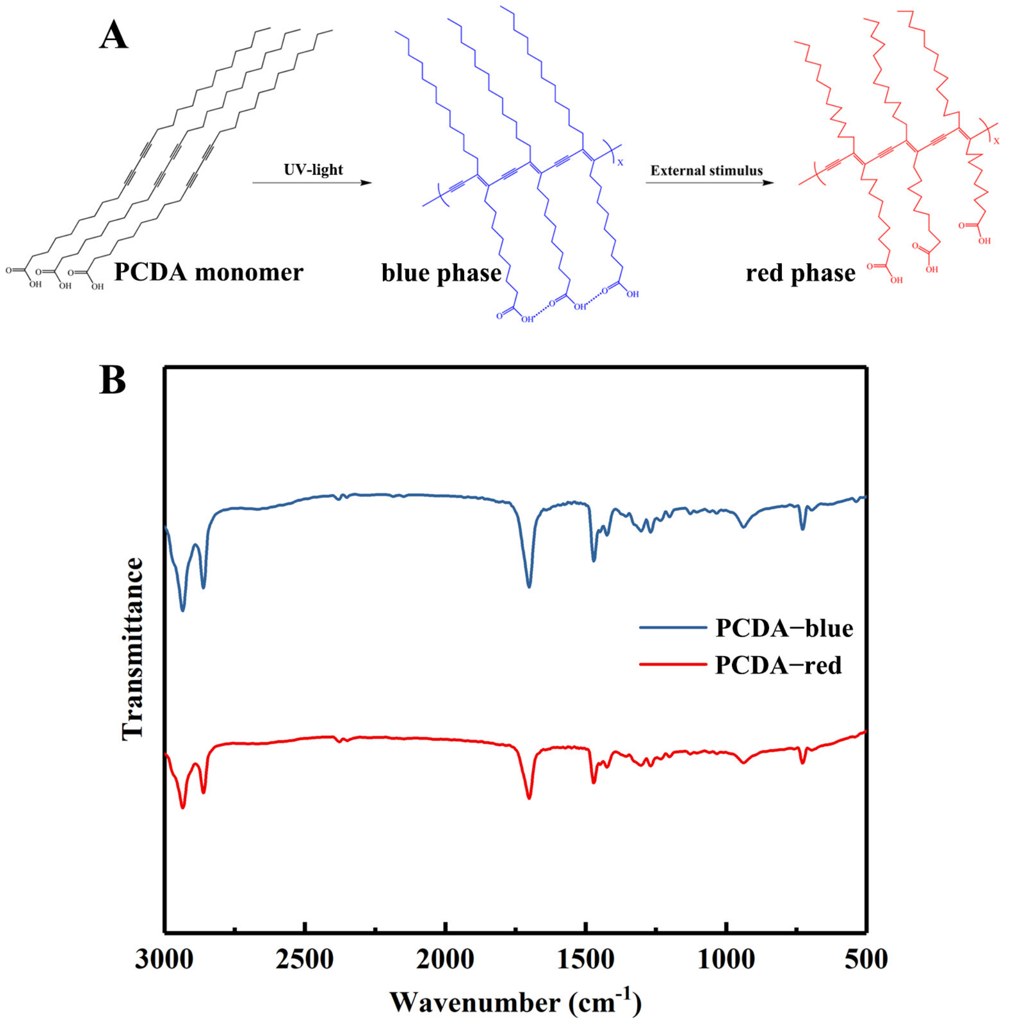

Figure S1. Chemical structures of PCDA at situations of monomer, blue phase and red phase (A), and the FT−IR curves of blue phase and red phase (B).

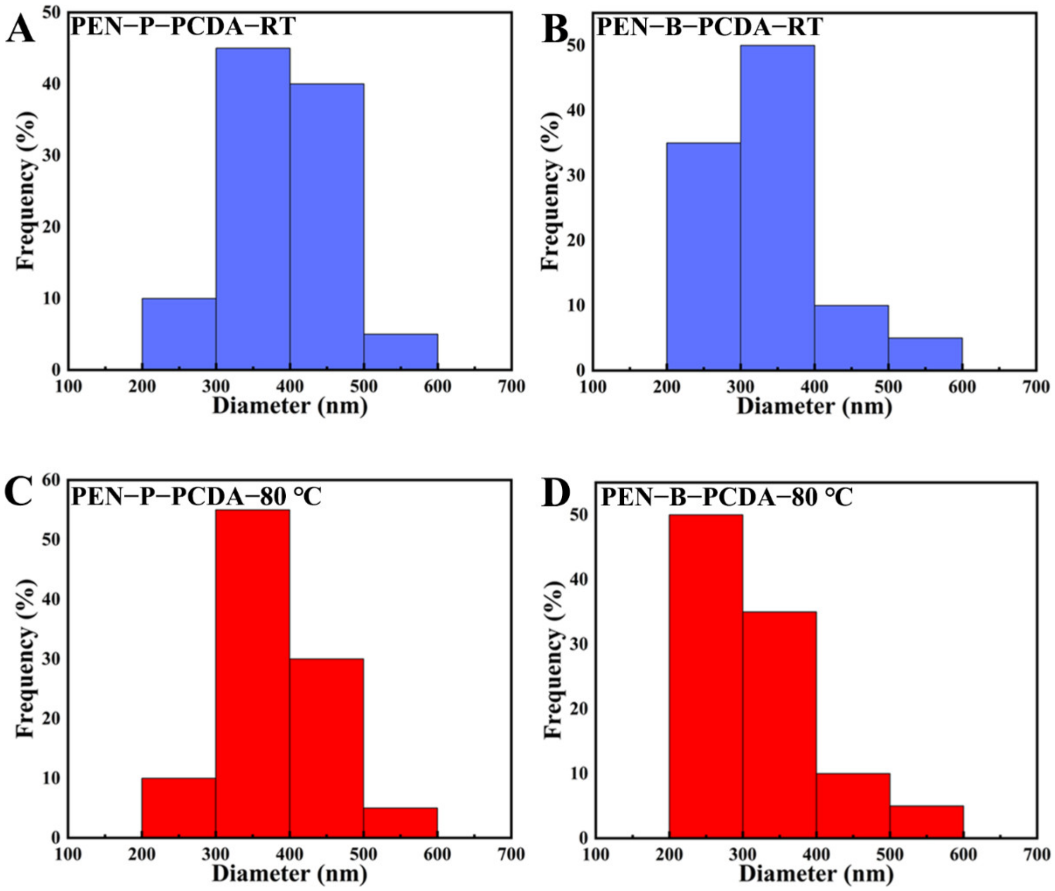

Figure S2. Statistical results of diameter of PEN−P−PCDA (A and C) and PEN−B−PCDA (B and D) nanofibrous membranes.
